# Deep-Penetrating and High-Resolution Continuous-Wave Nonlinear Microscopy Based on Homologous Dual-Emission Upconversion Adaptive Optics

**DOI:** 10.1021/acs.nanolett.5c01030

**Published:** 2025-03-20

**Authors:** Jing Yao, Zhipeng Yu, Yufeng Gao, Baoju Wang, Zhiyuan Wang, Tianting Zhong, Binxiong Pan, Huanhao Li, Hui Hui, Wei Zheng, Qiuqiang Zhan, Puxiang Lai

**Affiliations:** † Department of Biomedical Engineering, Hong Kong Polytechnic University, Hong Kong SAR 999077, China; ‡ Centre for Optical and Electromagnetic Research, Guangdong Engineering Research Centre of Optoelectronic Intelligent Information Perception, Guangzhou 510006, China; § Research Center for Biomedical Optics and Molecular Imaging, Shenzhen Key Laboratory for Molecular Imaging, Guangdong Provincial Key Laboratory of Biomedical Optical Imaging Technology, Shenzhen Institute of Advanced Technology, Chinese Academy of Sciences, Shenzhen 518055, China; ∥ Hong Kong Polytechnic University Shenzhen Research Institute, Shenzhen 518055, China; ⊥ Key Laboratory of Molecular Imaging, Institute of Automation, Chinese Academy of Sciences, Beijing 100190, China; # Photonics Research Institute, Hong Kong Polytechnic University, Hong Kong SAR 999077, China

**Keywords:** adaptive optics, upconversion nanoparticles, nonlinear fluorescence microscopy, continuous-wave excitation, wavefront shaping, deep-tissue imaging

## Abstract

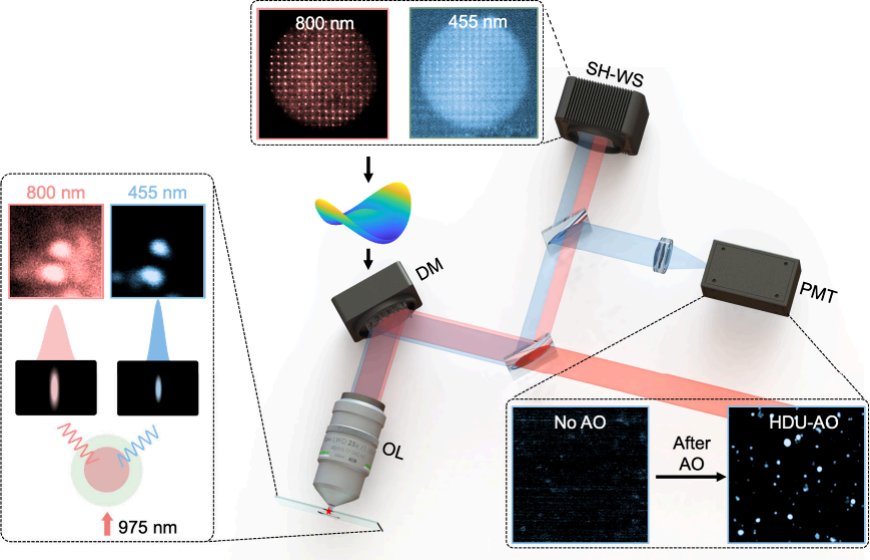

Lanthanide-doped upconversion nanoparticles (UCNPs) are emerging as innovative nonlinear probes in biomedical studies, offering the unique capability to simultaneously emit both visible (VIS) and near-infrared (NIR) photons under continuous-wave (CW) NIR excitation. However, deep-tissue high-resolution imaging remains challenging due to the trade-off between VIS emission (higher resolution, limited penetration) and NIR emission (deeper penetration, lower resolution). Here we present a CW nonlinear microscopy based on homologous dual-emission upconversion adaptive optics, leveraging Tm^3+^/Yb^3+^ co-doped UCNPs’ dual 455 nm/800 nm emission: the 800 nm emission for aberration measurement (guide-star) in deep tissues and the 455 nm emission for high-resolution imaging at matching depths. Using a home-built nonlinear laser scanning microscope with a 975 nm CW laser, we achieved near-diffraction-limited imaging (480 nm laterally) at a 500 μm depth in the mouse brain environment with significant optical aberrations. This strategy expands UCNPs’ applications and innovates the exploration of deep-tissue optical features.

Nonlinear optical microscopy (NLOM), exemplified by multiphoton microscopy (MPM),^1−5^ utilizes nonlinear light–matter interactions to generate fluorescence for imaging. Over the past 20 years, NLOM has been widely used to reveal cellular structures,^6,7^ biomolecular distributions,^8^ and the dynamics of life processes.^9−12^ In these applications, imaging resolution usually increases with the order of nonlinearity,^13^ which, however, is practically constrained, as conventional nonlinear fluorophores exhibit low-order nonlinearities due to their small absorption cross-section. Consequently, high-intensity ultrafast femtosecond laser pulses are indispensable for generating sufficient nonlinear signals.^14^ Additionally, some issues such as photobleaching, phototoxicity,^15^ and re-excitation background^16^ associated with the use of these nonlinear fluorophores cannot be ignored.

Upconversion nanoparticles (UCNPs), typically doped with ytterbium sensitizer ions (Yb^3+^), have been recently employed as novel probes in NLOM.^17−23^ Compared with traditional nonlinear fluorophores, UCNPs possess longer lifetime of the energy levels,^24,25^ enabling a sequential photon absorption process that converts high-energy NIR excitation into multiple anti-Stokes emissions.^26−28^ This bypasses the aforementioned nonlinearity excitation requirements, allowing for the use of more cost-effective and readily available continuous-wave (CW) NIR lasers instead of high-intensity femtosecond lasers. Furthermore, UCNPs inherently exhibit higher-order nonlinearity, providing higher resolution and better signal-to-noise ratio (SNR) than traditional nonlinear fluorophores.^29−31^

Despite these advantages of UCNPs and the use of NIR laser excitation to mitigate scattering, aberrations still persist in deep tissue,^32^ similar to those encountered in conventional MPM.^33^ In deep tissue imaging, optical aberrations and scattering disrupt the formation of a diffraction-limited focus, thereby diminishing signal integrity, contrast, and resolution.^34−38^ Traditionally, a direct-wavefront-sensing adaptive optics (AO) method^39−42^ has been developed to recover to the diffraction-limit focus by correcting aberrations along the excitation path in scattering media. This method employs a Shack–Hartman wavefront sensor (SH-WS) to measure wavefront distortion by creating a fluorescent guide star (GS) inside the specimen. The clarity of the GS image across SH-WS elements is essential for effective aberration correction.^43^ However, in tissues such as the mammalian brain, strong scattering attenuates the GS-forming ballistic fluorescence and generates a diffuse background that can obscure the ballistic signal, complicating aberration measurement. Optical scattering is wavelength-dependent, with shorter wavelengths experiencing more scattering. Consequently, while the visible (VIS) GS of the UCNPs offers improved imaging quality due to their high-order nonlinearity, it is not optimal for aberration measurements in deep tissues because of its susceptibility to scattering.

Here, we proposed a deep-penetrating and high-resolution CW nonlinear microscopy based on homologous dual-emission upconversion adaptive optics (HDU-AO) by employing the homologous dual-emission feature of the Tm^3+^ and Yb^3+^ co-doped UCNPs upon CW excitation at 975 nm. The NIR (800 nm) emission of UCNPs serves as the GS due to its reduced scattering and its wavefront closer alignment with the excitation light’s distortion profile, and the VIS (455 nm) emission is utilized for high-resolution imaging owing to its four-photon upconverting effect. Through comparison, it reveals that the 455 nm emission offers superior resolution and SNR, whereas the 800 nm emission provides the capability to penetrate more deeply within the mouse brain environment. Subsequently, we demonstrated the applicability of the proposed method to in vitro imaging, clearly resolving nanoparticles with a lateral resolution of 480 nm within a complex 500 μm-thick mouse brain environment by integrating our approach into a home-built nonlinear laser scanning microscope.

The detailed procedure of HDU-AO is illustrated in Figure 1. The UCNPs, co-doped with Tm^3+^ and Yb^3+^, are designed to emit two distinct upconverted wavelengths: VIS at 455 nm and NIR at 800 nm upon excitation with a 975 nm CW laser. As shown in Figure 1a, the VIS emission exhibits a much higher nonlinear effect compared with the NIR emission, resulting in a sharper point spread function (PSF), which is chosen for imaging. Conversely, the NIR emission is used as the GS for aberration correction because it experiences less scattering and provides a significantly closer alignment with the distortion profile of the excitation light than the VIS emission. This results in a much clearer and brighter image consisting of a spot matrix captured by the SH-WS (Figure 1b). By leveraging the 800 nm emission as the GS for correction in deeper tissues, we can achieve high-resolution AO imaging using the 455 nm emission, even at challenging depths (Figure 1c).

**Figure 1:**
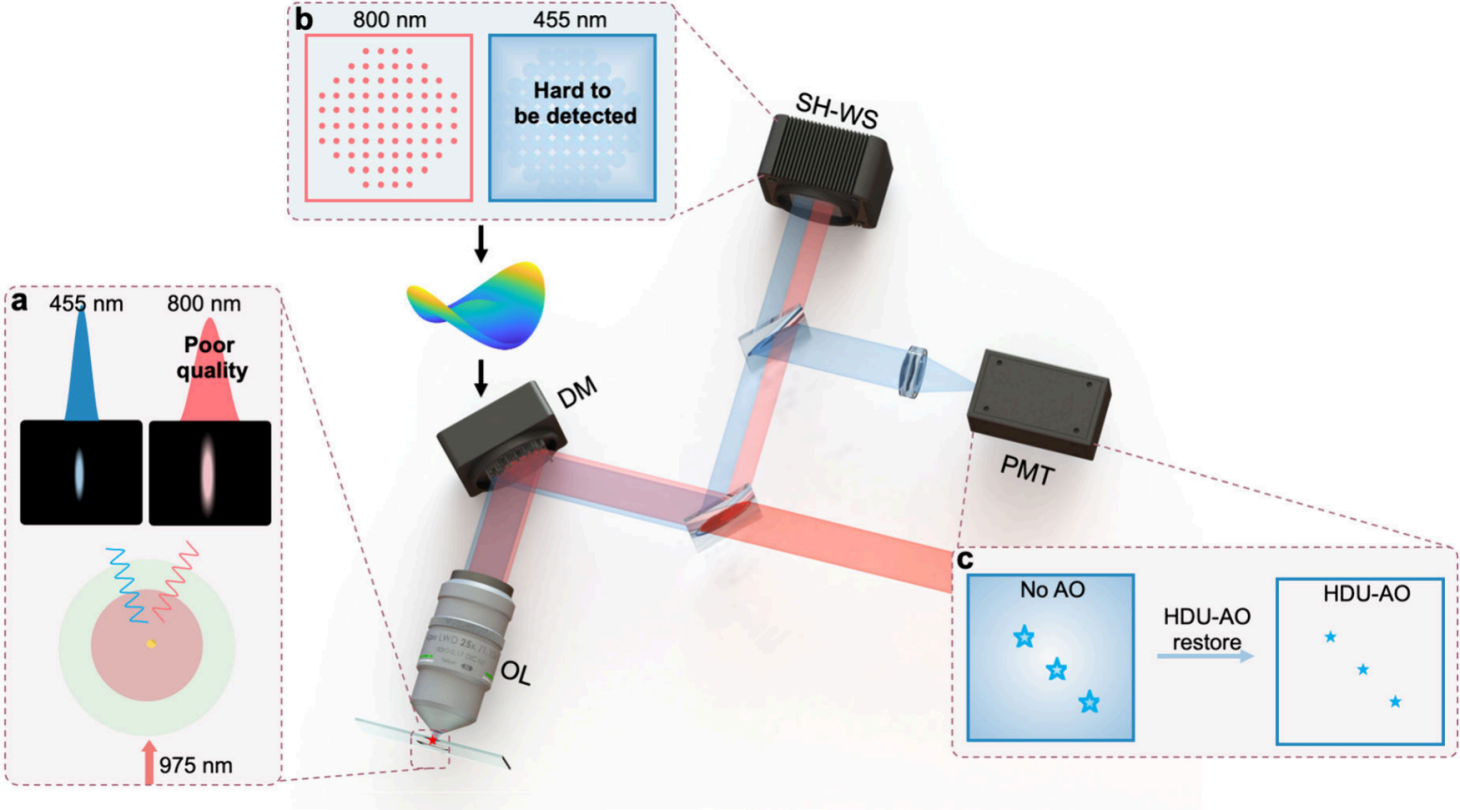
Principle of the proposed HDU-AO microscopy. (a) The emission characteristic of Tm^3+^ and Yb^3+^ co-doped UCNPs; (b) illustrative spot diagrams captured by the SH-WS; (c) illustrative images captured by the photomultiplier tube (PMT).

The UCNPs utilized in this study are co-doped with Tm^3+^ and Yb^3+^, exhibiting the remarkable property of emitting dual emissions at both 455 and 800 nm when excited by a 975 nm CW laser. These particles are characterized by uniform morphologies with an average size of 33 nm, as evidenced by the transmission electron microscopy (TEM) image presented in Figure 2a. As depicted in Figure 2b, when excited by a 975 nm CW laser, the Yb^3+^ and Tm^3+^ ions successively absorb multiple photons, transitioning from ground state ^2^F_7/2_ to excited states ^1^D_2_ and ^3^H_4_, subsequently reaching the ^3^F_4_ and ^3^H_6_ states, which are responsible for the emissions at 455 and 800 nm, respectively. In the proposed HDU-AO, we aim to utilize both the 455 and 800 nm emissions effectively. As illustrated in Figure 1, by precompensating the aberrations through the 800 nm emission wavefront sensing, an aberration-free focus is capable of exciting the 455 nm fluorescence signals, which can then be detected by a PMT. Through our experiments, we have identified an optimal concentration of Tm^3+^ doping. This specific concentration allows us to balance the intensities of both the 455 and 800 nm emissions, ensuring their effective use in HDU-AO (Figure 2c). Figure 2d shows the nonlinear optical response behaviors of the UCNPs. The results demonstrate that the emission at 800 nm corresponds to a two-photon excitation process (Slope_800_ = 1.69), while the emission at 455 nm results from a four-photon excitation process (Slope_455_ = 4.03). The 800 nm emission demonstrates superior excitation efficiency over the 455 nm emission at lower power densities, enabling effective laser excitation and subsequent detection via SH-WS under less strict excitation conditions.

**Figure 2:**
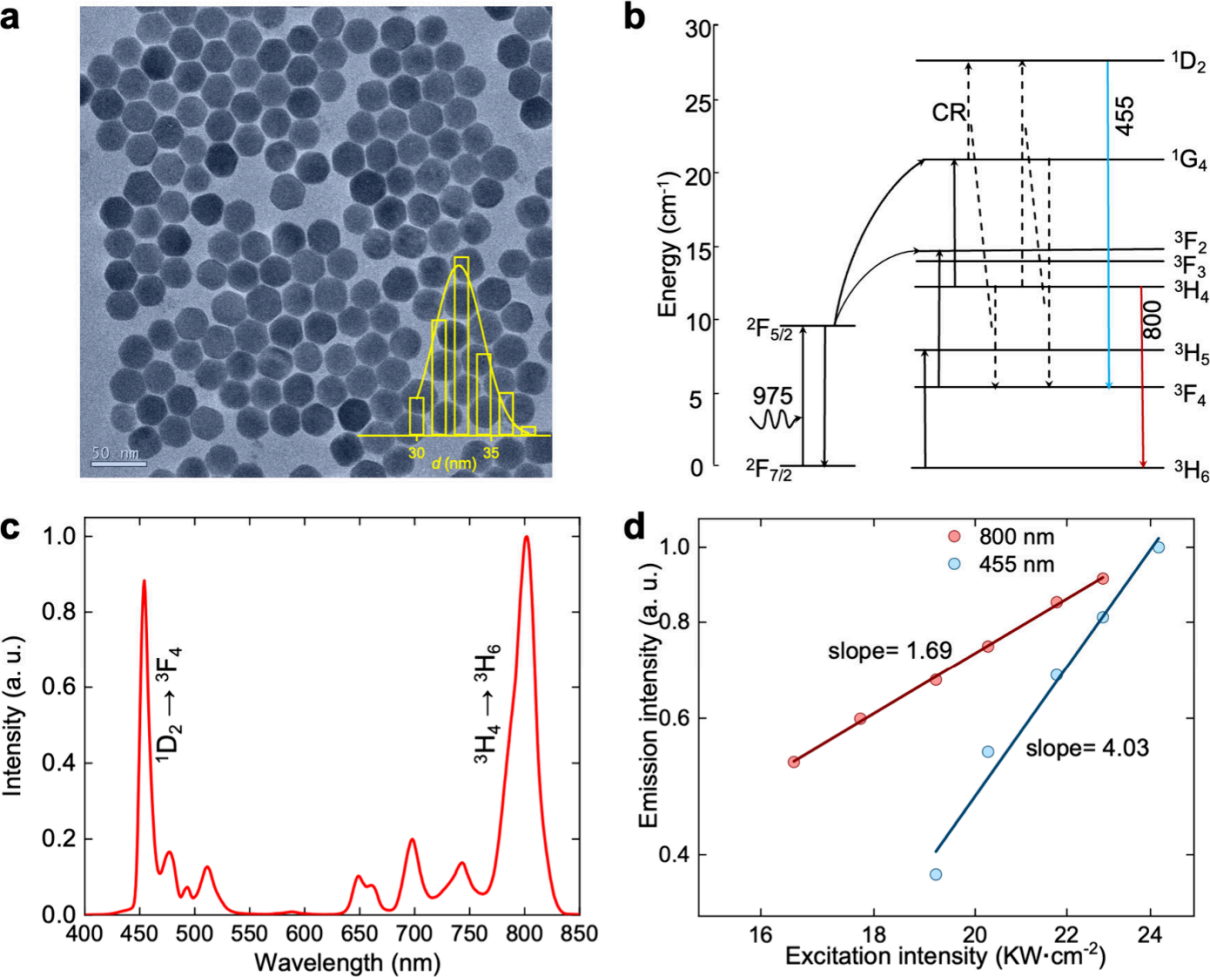
Characteristics of the utilized UCNPs co-doped with Tm^3+^ and Yb^3+^. (a) TEM images of utilized UCNPs, whose average size is around 33 nm. Scale bar, 50 nm. (b) The energy diagram and upconversion process of Yb^3+^ and Tm^3+^ co-doped UCNPs. The sensitizer Yb^3+^ ions initiate the photon upconversion process by the 975 nm excitation. The NIR upconversion emissions are mainly composed of the two-photon excited state (800 nm, ^3^H_4_ → ^3^H_6_) and four-photon excited state (455 nm, ^1^D_2_ → ^3^F_4_). (c) The upconversion emission spectrum of UCNPs under 975 nm excitation. (d) The excitation intensity-dependent emission curve, Slope_455_ = 4.03, Slope_800_ = 1.69.

A comparative analysis of the imaging quality and penetration capability of the VIS and NIR emissions is conducted, as shown in Figure 3. The scanning microscopic images of the UCNPs (without a scattering cover), using VIS and NIR emissions, are presented in Figure 3a. The results of the 800 nm emission exhibit significant background noise when compared to those of the 455 nm emission, as shown in Figure 3a. This is primarily due to the 800 nm emission being associated with a two-photon excitation process (Slope_800_ = 1.69, Figure 2d), which leads to a considerable out-of-focus fluorescence background. Furthermore, the resolutions of the two UC beads presented in Figure 3a(iii) and (iv) show a notable improvement, with the resolutions enhancing from 990 and 850 nm to 610 and 590 nm, respectively. These results indicate that the VIS emission provides a substantially higher SNR and superior resolving power (Figure 3a), suggesting that the VIS emission is more suitable for high-resolution imaging. To assess the penetration capability, the UCNP sample is covered with various mouse brain slices from BALB/c mice (Figure 3b), with thickness ranging from 100 to 600 μm in increments of 100 μm (Figure S2). As the thickness of the brain slice increases, images captured by the SH-WS using VIS and NIR emission are shown in Figures 3c and 3d, respectively. The results demonstrate that the VIS emission’s spot diagrams become blurred and indistinguishable, particularly at a thickness beyond 300 μm, while the spot diagrams of the NIR emission remain clearly distinguishable all through the experiments. This indicates that the NIR emission is more appropriate for use as the GS. Because VIS and NIR emissions are homologous, HDU-AO provides a robust and straightforward strategy to achieve high-resolution imaging in deep tissue under CW excitation.

**Figure 3:**
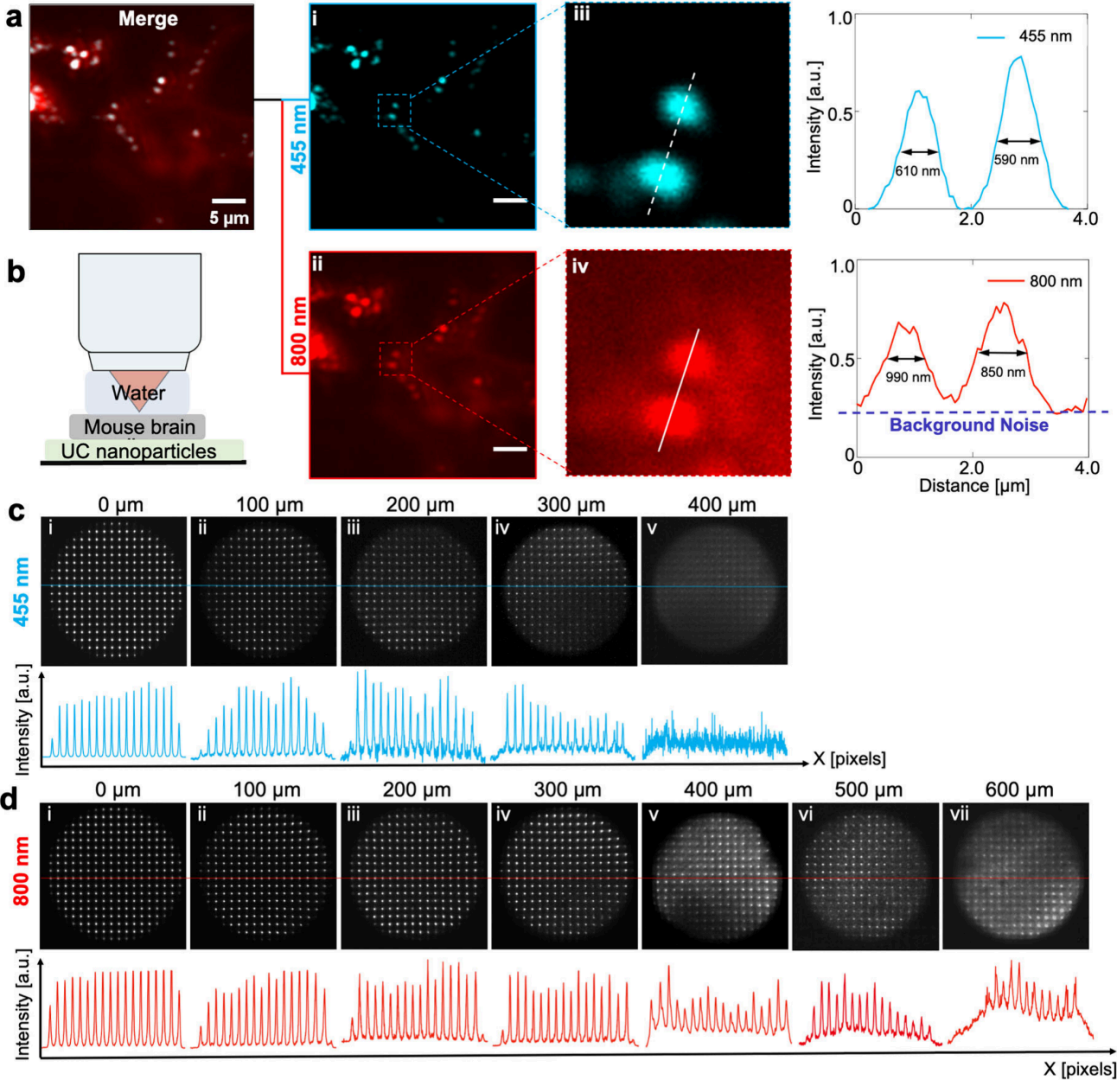
Comparison of imaging with the 455 nm (VIS) and 800 nm (NIR) emissions. (a) Imaging quality comparison when there is no scattering cover above the UCNPs. (b) Depiction of the sample arrangement for comparison of penetration capability, with mouse brain slices of various thickness to cover the UCNPs. (c, d) Spot diagrams captured by the SH-WS through mouse brain slices of different thickness for 455 nm (c) and 800 nm (d) emissions, respectively. The blue curves in (c) and the red curves in (d) are the representative horizontal profile of the corresponding spot diagram.

The aberration from the refractive mismatch is first investigated using the proposed method. Cuboid and oblique polydimethylsiloxane (PDMS) pieces (refractive index: 1.41) are individually positioned between the objective lens and the sample, resulting in significant refractive mismatch (Figures 4a and 4g). The spot diagrams captured by the SH-WS for each PDMS type (Figures 4e and 4k) are analyzed, and the aberrations are subsequently reconstructed using spot-shift diagrams and Zernike polynomials (Figures 4f and 4l).^44^ The calculated results reveal that the cuboid PDMS configuration primarily induces spherical aberration (Figure 4f), while the oblique configuration introduces substantial coma and astigmatism (Figure 4l). Here we acquired the images at an ∼25 kW/cm^2^ excitation power density. After correction, significant enhancements in image sharpness and clarity for nanoparticles are observed (Figures 4b and 4h). Notably, nanoparticles with sharp deformation due to asymmetrical aberrations regain their normal circular shape after correction (Figure 4h). To demonstrate the efficacy more concretely, one-bead images from three different perspectives (Figures 4c and 4i) are extracted from the full field of view (FOV). As seen, after correction, unwanted ghost signals are effectively eliminated, resulting in significant improvement in both lateral and axial resolution, as well as the peak signal intensity, as depicted in Figures 4d and 4j, which show the lateral (i) and axial (ii) intensity profiles for each PDMS type, respectively.

**Figure 4:**
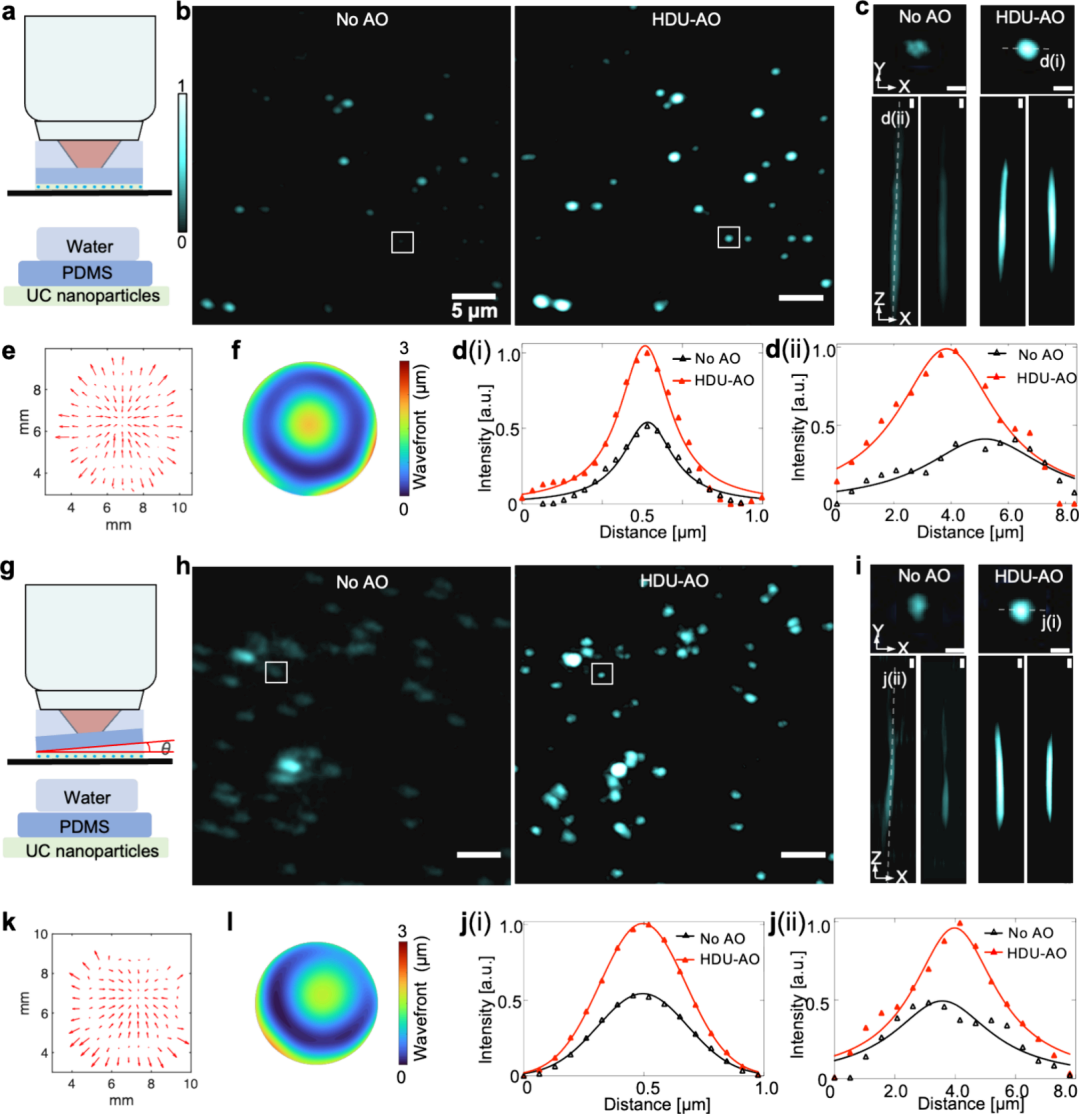
HDU-AO compensation for aberrations induced by refractive mismatch of the PDMSs. (a, g) Sample configurations for two variants of PDMS. (b, h) Max intensity project (MIP) image comparisons of aberration corrections without and with AO. (c, i) Three views of a single bead image extracted from the whole-FOV images. (d, j) Intensity profile comparisons along the indicated lines in (c) and (h). (e, k) Spot shift diagrams corresponding to the reference aberration-free pattern. (f, l) Reconstructed wavefront patterns based on the Zernike modes. The scale bars in this figure represent 5 μm in large images and 1 μm in zoom-in images, respectively.

To evaluate the performance of our HDU-AO method in a deep tissue environment, we performed imaging of a sample covered by a 500 μm-thick fixed mouse brain slice obtained from BALB/c mice (Figure 5a). Without correction, the excitation wavefront is severely distorted by the brain slice, making the signal indistinguishable from the background noise (Figure 5b). As seen, the aberration correction markedly enhanced the visibility and resolution, as evidenced by the comparison of color-coded lateral images in Figure 5b. The spot-shift diagrams and aberration patterns revealed that the scattering of a 500 μm brain slice mainly resulted in spherical aberration (Figures 5c(i) and c(ii)). Here we acquired the images at ∼60 MW excitation power before the 500 μm mouse brain slice. To quantitatively validate the aberration correction ability, we selected images at two distinct depths (522 and 530 μm) (Figures 5d–g). Notably, the magnified images from four subregions at both 522 and 530 μm depths show a pronounced enhancement in signal and resolution (Figures 5d–g). The distorted foci at the focal plane of the objective lens caused by tissue scattering severely degraded the excitation of the VIS signals, leading to diffuse morphology for some large nanoparticles (Figure 5d(ii) and f(ii)) and a failure to excite the majority of nanoparticles (Figure 5d(iii) and f(iii)). After correction, VIS signals can be accurately excited, providing high resolution and high SNR with peak intensity enhanced by more than 5-fold (Figure 5h). Besides, the Fourier spectra shown in Figures 5d–g(iv) reveal that, after HDU-AO correction, more high-frequency components become discernible. Especially, both the Fourier spectra and the intensity profiles in Figures 5h(ii) and 5h(iv) indicate that the resolution reaches approximately 480 nm, resembling the diffraction-limited imaging in the absence of strong scattering. These results suggest that HDU-AO can achieve high-resolution imaging with VIS emission in deep tissue after aberration correction with NIR emission as the guide star for AO.

**Figure 5:**
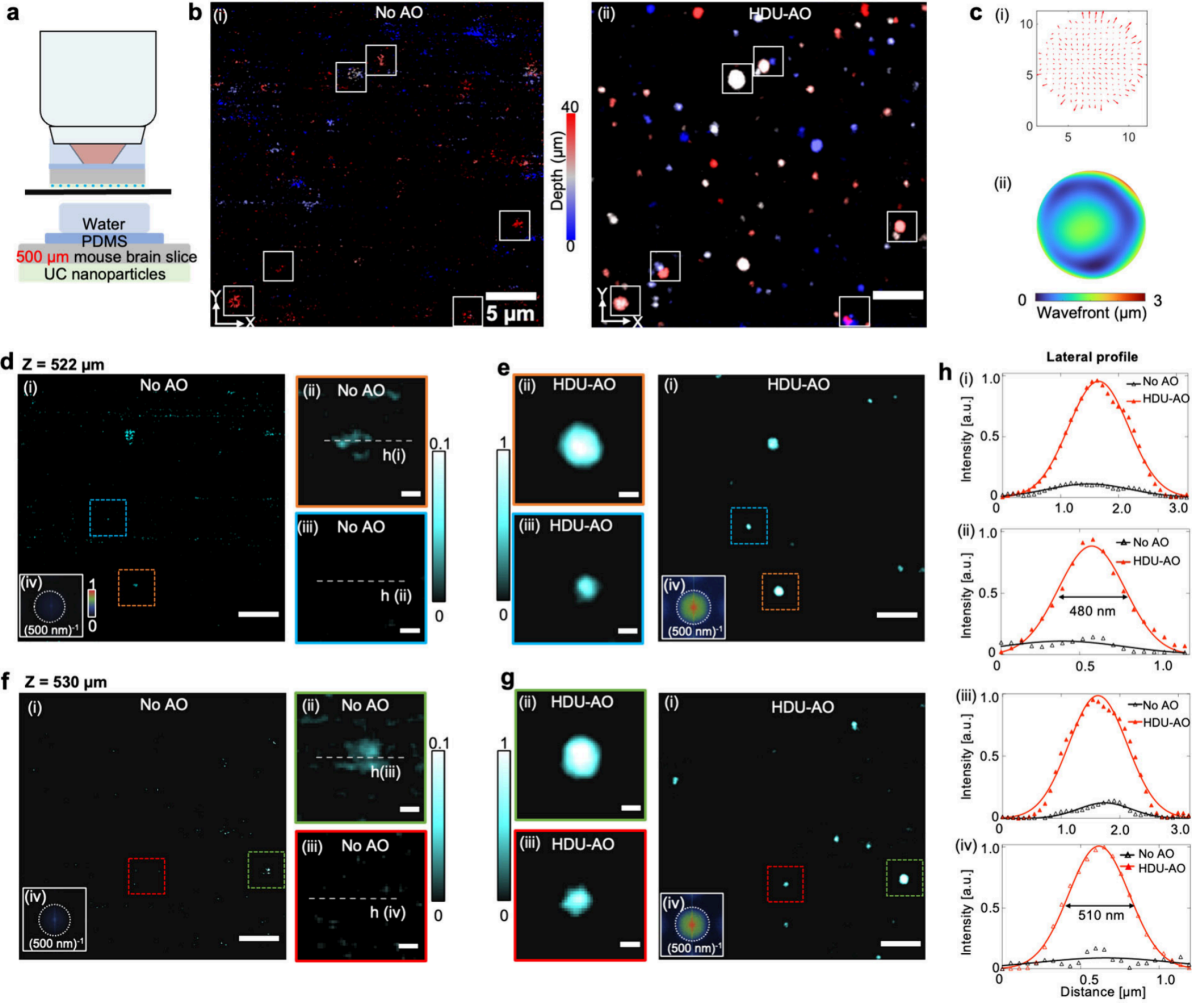
HDU-AO imaging in deep tissue (through mouse brain). (a) Sample configuration. (b) Comparison of 33 μm × 33 μm × 40 μm volumes of 20 slices with and without HDU-AO, color-coded by depths. (c) Spot shift diagrams corresponding to the reference aberration-free pattern and the wavefront patterns restructured by Zernike modes. (d–g) Images of two different depths with and without HDU-AO. (h) Intensity profile comparisons along the indicated lines in (d–g). The scale bars in this figure represent 5 μm in large images and 500 nm in zoom-in images, respectively.

In this study, we introduced a CW nonlinear imaging microscopy based on homologous dual-emission upconversion adaptive optics, a novel approach to achieve high resolution in deep tissues that leverages Tm^3+^ and Yb^3+^ co-doped UCNPs capable of emitting at both 455 and 800 nm simultaneously under a 975 nm CW excitation. This dual-emission feature of UCNPs offers a new perspective for measuring and correcting aberration to achieve high-resolution imaging in deep tissue. By harnessing the homologous dual-emission feature and other advantages of UCNPs, this innovative configuration effectively overcomes traditional AO limitations in deep tissues, achieving a resolution of approximately 500 nm with minimal background signal across depths of at least 500 μm through a brain slice.

Furthermore, a significant advantage of UCNPs for the AO technique in achieving high-resolution deep tissue imaging is their flexible multiple emissions through diverse ion-doping,^45^ thus offering a wide range of options for optimizing the imaging depth and resolution in various applications. That said, the primary challenge in direct-wavefront-sensing AO predominantly arises from the difficulty in capturing clear GS images deep within biological tissue. We anticipate that the imaging depth can be further extended by utilizing GS-used emissions at an even longer wavelength, which can be excited by the upconversion effect or downconversion effect^46^ with various ion doping, thereby enhancing the detectability of wavefront sensing.

Beyond the outstanding resolution in challenging depths, a standout feature of this technique is the low light toxicity provided by CW laser excitation. This significantly diminishes photobleaching and photodamage due to low-power CW laser excitation, enabling long-term dynamic tracking with wavelength-scale resolution in deep tissue. Additionally, this technique can be synergistically integrated with other microscopy techniques as an add-on feature to enhance penetration depth while maintaining resolution using only a low-cost CW laser excitation. This exceptional extensibility offers significant convenience for different tissue imaging requirements, such as finer resolution or deeper penetration depth.

Overall, the HDU-AO method provides a robust solution for high-resolution imaging in deep tissue. This technique holds great promise for a wide range of biomedical applications. It allows for detailed and long-term monitoring of biological structures with low-cost and low-toxicity CW excitation, enabling researchers to explore the intricacies of biological structures with unprecedented clarity and precision at the most challenging depth.

## Supporting Information Available

Materials and methods: synthesis of UCNPs, experimental setup, tissue sample preparation, the principle of HDU-AO; supplementary figures: the optical setup of the proposed method, mouse brain slices with different thickness (PDF)
