# Supplementary material for: Deep-Penetrating and High-Resolution Continuous-Wave Nonlinear Microscopy Based on Homologous Dual-Emission Upconversion Adaptive Optics
Source: Nano Lett. 2025 Mar 20;25(13):5485–92. doi: 10.1021/acs.nanolett.5c01030 (PMC11969653; doi:10.1021/acs.nanolett.5c01030)
Supplement: Supplementary file 1 — nl5c01030_si_001.pdf [file nl5c01030_si_001.pdf]

Deep-penetrating and high-resolution continuous-wave nonlinear microscopy based on homologous dual-emission upconversion adaptive optics

Jing Yao[a,c,d§], Zhipeng Yu[a,d§], Yufeng Gao[c§], Baoju Wang[b§], Zhiyuan Wang[a,d], Tianting Zhong[a,d], Binxiong Pan[b], Huanhao Li[a,d], Hui Hui[e*], Wei Zheng[c*], Qiuqiang Zhan[b*], and Puxiang Lai[a,d,f*]

[a] Department of Biomedical Engineering, Hong Kong Polytechnic University, Hong Kong SAR 999077, China.

[b] Centre for Optical and Electromagnetic Research, Guangdong Engineering Research Centre of Optoelectronic Intelligent Information Perception, Guangzhou 510006, China.

[c] Research Center for Biomedical Optics and Molecular Imaging, Shenzhen Key Laboratory for Molecular Imaging, Guangdong Provincial Key Laboratory of Biomedical Optical Imaging Technology, Shenzhen Institute of Advanced Technology, Chinese Academy of Sciences, Shenzhen 518055, China.

[d] Hong Kong Polytechnic University Shenzhen Research Institute, Shenzhen 518055, China.

[e] Key Laboratory of Molecular Imaging, Institute of Automation, Chinese Academy of Sciences, Beijing 100190, China.

[f] Photonics Research Institute, Hong Kong Polytechnic University, Hong Kong SAR 999077, China.

[§] These authors contributed equally.

*Corresponding e-mail: puxiang.lai@polyu.edu.hk; zhanqiuqiang@m.scnu.edu.cn; zhengwei@siat.ac.cn; hui.hui@ia.ac.cn;

Supplementary Note 1 Synthesis of UCNPs

Purchase of materials. Sodium hydroxide (NaOH, methanol solution, ≥99.9%), ammonium fluoride (NH_4F, methanol solution, ≥99.9%), oleic acid (OA, analytical reagent (AR)) and 1-octadecene (ODE, ≥90% (GC)) were purchased from Aladdin. Yttrium (Y, acetate hydrate, 99.9%), thulium (Tm, acetate hydrate,99.9%), ytterbium (Yb, acetate hydrate, 99.9%) were purchased from Sigma-Aldrich. Methanol (reagent grade), ethanol (reagent grade) and cyclohexane (reagent grade) were purchased from Sinopharm Chemical Reagent.

Sodium hydroxide (NaOH, methanol solution, ≥99.9%), ammonium fluoride (NH_4F, methanol solution, ≥99.9%), oleic acid (OA, analytical reagent (AR)) and 1-octadecene (ODE, ≥90% (GC)) were purchased from Aladdin. Yttrium (Y, acetate hydrate, 99.9%), thulium (Tm, acetate hydrate,99.9%), ytterbium (Yb, acetate hydrate, 99.9%) were purchased from Sigma-Aldrich. Methanol (reagent grade), ethanol (reagent grade) and cyclohexane (reagent grade) were purchased from Sinopharm Chemical Reagent.

Synthesis of $NaYF_4$:Yb/Tm (50/4%) core nanoparticles. The designed lanthanide-doped upconversion nanoparticles were synthesized based on a protocol reported previously with some modifications[1]. Initially, added a 5 mL portion of an aqueous solution of $Ln(CH_3CO_2)_3$, containing 0.04 mmol Tm^{3+}, 0.46 mmol Y^{3+}, and 0.5 mmol Yb^{3+}, to a 100 mL round-bottom flask that already held 7.5 mL OA and 17.5 mL ODE. The stirring mixture was heated to 120°C for 10 minutes to eliminate water from the solution, then continued heating to 150°C for 40 minutes to form lanthanide-oleate precursor. After naturally cooling to 40°C, 10 mL NH_4F (0.4 mmol/mL in methanol) and 2.5 mL of NaOH (1 mmol/mL in methanol) were added into the flask. Next, stirred the mixture at 40°C for at least two hours to clarify it, then heated the solution

under vacuum to 100°C for 30 minutes to eliminate methanol, followed by filling the flask with argon gas. The mixture was heated to 300°C for 1.5 hours under anhydrous and anaerobic conditions, then cooled down to the room temperature. To precipitate nanoparticles, added 15 mL anhydrous ethanol to the mixture, and then collected the nanoparticles by centrifuging at 7500 r.p.m. (relative centrifugal force (RCF) = 4788×g) for 5 minutes. The obtained nanoparticles were washed several times with anhydrous ethanol and cyclohexane, and finally re-dispersed them into 8 mL cyclohexane for subsequent use.

Synthesis of $NaYF_4$:Yb/Tm@$NaYF_4$ core-shell nanoparticles. The synthesis of the $NaYF_4$ shell is analogous to that of the $NaYF_4$:Yb/Tm (50/4%) core[2]. A 1 mmol solution of $Y(CH_3CO_2)_3$ (0.2M), 7.5 mL OA, and 17.5 mL ODE was put into a 100 mL three-necked flask. Then heated the mixture to 150°C with stirring for 50 minutes. Subsequently, upon cooling the flask to 90°C, 4 mL of the prepared $NaYF_4$:Yb/Tm (50/4%) core was injected into the flask. The subsequent steps were identical to those for the synthesis of the $NaYF_4$:Yb/Tm (50/4%) core, except that the core-shell nanoparticles were ultimately stored in 4 mL cyclohexane.

Supplementary Note 2 Experimental Setup

The experiment setup of HDU-AO includes a nonlinear imaging module, an SH-WS, and a wavefront correction device (Fig. S1). Briefly, the excitation light (975 nm) from a laser (OS8147-975-900, B&A technology Co., Ltd, and Chameleon Ultra, Coherent) was expanded ten folds by a lens pair (L1: AC254-30-B, L2: AC254-300-B, Thorlabs) to overfill the aperture of a deformable mirror (DM, DM140A-35-P01, Boston Micromachine). After the DM, the excitation beam was raster scanned by a pair of galvanometer mirrors (Galvo XY, TS8203, Sunny Technology) and then focused onto the samples by a water-immersion objective (N16XLWD-PF Nikon, 25XAPO-MP Nikon). Note that the surfaces of the DM, Galvo X&Y,

and the back pupil of the objective were made mutually conjugated through three pairs of relay lenses operating in the 4f configuration (L3: AC254-300-B, L4: AC254-250-B; L5: AC508-080-AB and AC508-080-AB lenses coupled; L6: two AC508-080-AB lenses coupled, Thorlabs). For nonlinear imaging, the fluorescence was collected by the objective and then reflected by a dichroic mirror DMBS (T715LP, Chroma) and focused by another lens pair (L7: LA1145-A, L8: LA1805-A, Thorlabs) onto a photomultiplier tube (PMT, H7422P-40, Hamamatsu). A bandpass filter (MV450/20 nm, Chroma) was placed before the PMT to purify the fluorescence detection.

For aberration correction, the NIR fluorescence from the GS was descanned by Galvos X and Y, separated from the excitation light by a dichroic mirror BS2 (FF875-Di01-25x36, Semrock), passed through a beam expander in 4-f configuration (AC254-100-AB, AC254-200-AB, Thorlabs), purified by a filter wheel (FWM12S, LBTEK), and the fluorescence filter was switchable between VIS BS3 (FF01-720/SP-25, Semrock) and NIR filter (FF01-792/64-25, Semrock), and finally detected by the SH-WS. The SH-WS consisted of a microlens array (#64–483, Edmund Optics) conjugated with the objective rear pupil and a camera (Dhyana 400BSI, Tucsen) placed at the focal plane of the microlens array.

Besides, to compare the imaging performance of the 455%nm and 800%nm emissions, we split the light directed to the SH-WS into two paths by a beam splitter BS_3 (BS2555-T3M, JCOPTIX): one to the SH-WS and the other to a NIR-specific PMT detector (H7422P-40, Hamamatsu, peak sensitivity wavelength: 800%nm) equipped with a filter (FF01-792/64-25, Semrock). It should also be noted that BS1 was removed from the light path through a switch (ELL6K, Thorlabs) when measuring the penetration depth of the 455 nm emission.

A customized MATLAB code based on the ScanImage program[3] was used to capture the nonlinear excitation images, measure and compensate for the optical aberration, and control the devices. The SH-WS and the DM were synchronized via a MATLAB program. A National Instruments DAQ card (PCI6110) was used to control and synchronize the system as well as digitize the obtained fluorescence signals.

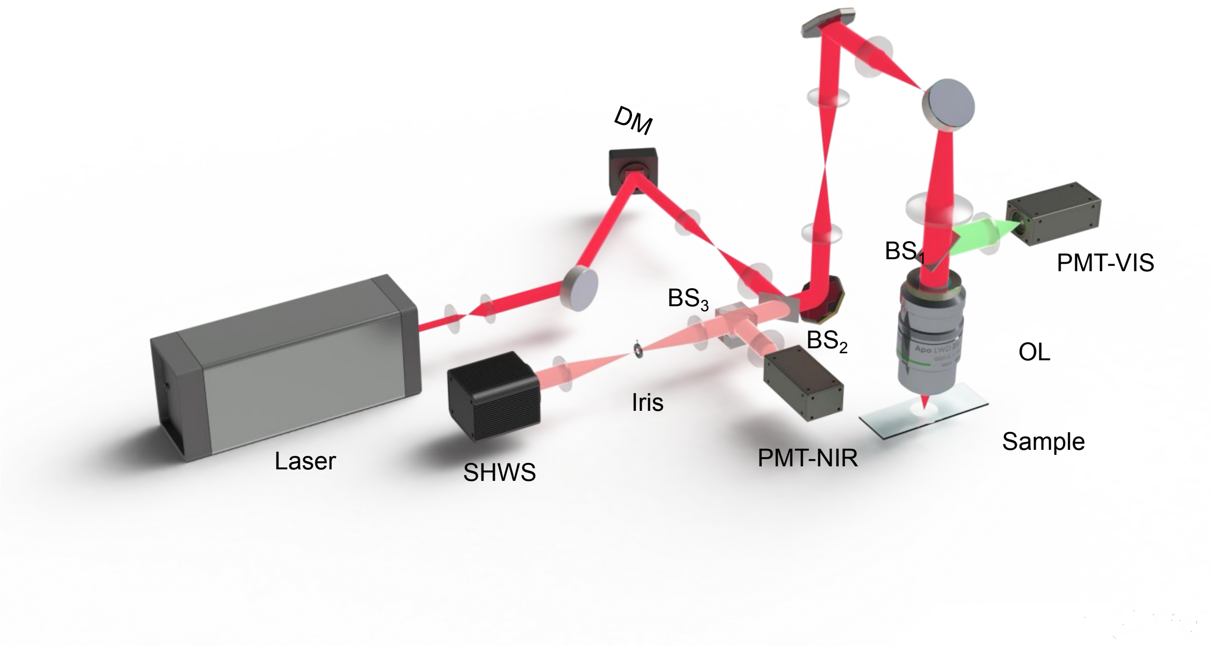

Fig. S1 The optical setup of proposed method. BE: beam expander; M: mirror; DM: deformable mirror; L: lens; BS: beam splitter; X-S: x-scanner; Y-S: y-scanner; PMT: Photomultiplier tube; OL: objective lens.

Supplementary Note 3 Tissue sample preparation

BALB/c mice aged 8-12 weeks were used for the experiment. All experiments were performed in compliance with the protocols approved by the Guangdong Provincial Animal Care and Use Committee and following the guidelines of the Animal Experimentation Ethics Committee of Shenzhen Institutes of Advanced Technology, Chinese Academy of Sciences. Mice were first anesthetized using an intraperitoneal injection of a ketamine-xylazine mixture to ensure deep sedation. Following anesthesia, the mice were euthanized through cervical dislocation as per ethical standards. The brain was immediately extracted from the skull using fine dissection tools, taking care not to damage the tissue. The extracted brain was then rinsed in cold phosphate-buffered saline to remove any remaining blood. Once extracted, the brain was embedded in Optimal Cutting Temperature compound to prepare for cryosectioning. The embedding step involved positioning the brain in the desired orientation for sectioning, followed by freezing the brain block using dry ice or storing it at -80˚C until the tissue was fully frozen. Once the brain was fully solidified, it was placed in a cryostat, which was pre-cooled to a temperature between -20˚C to -30˚C to ensure optimal cutting conditions.

The frozen brain tissue was then sectioned into slices of varying thickness using the cryostat. Brain slices of 100 μm, 200 μm, 300 μm, 400 μm, 500 μm, and 600 μm were prepared, as depicted in Figure S2. Each slice was carefully collected and transferred onto pre-labeled glass slides to preserve the integrity of the brain structure. The slides were stored at -20˚C or colder until they were ready for further analysis.

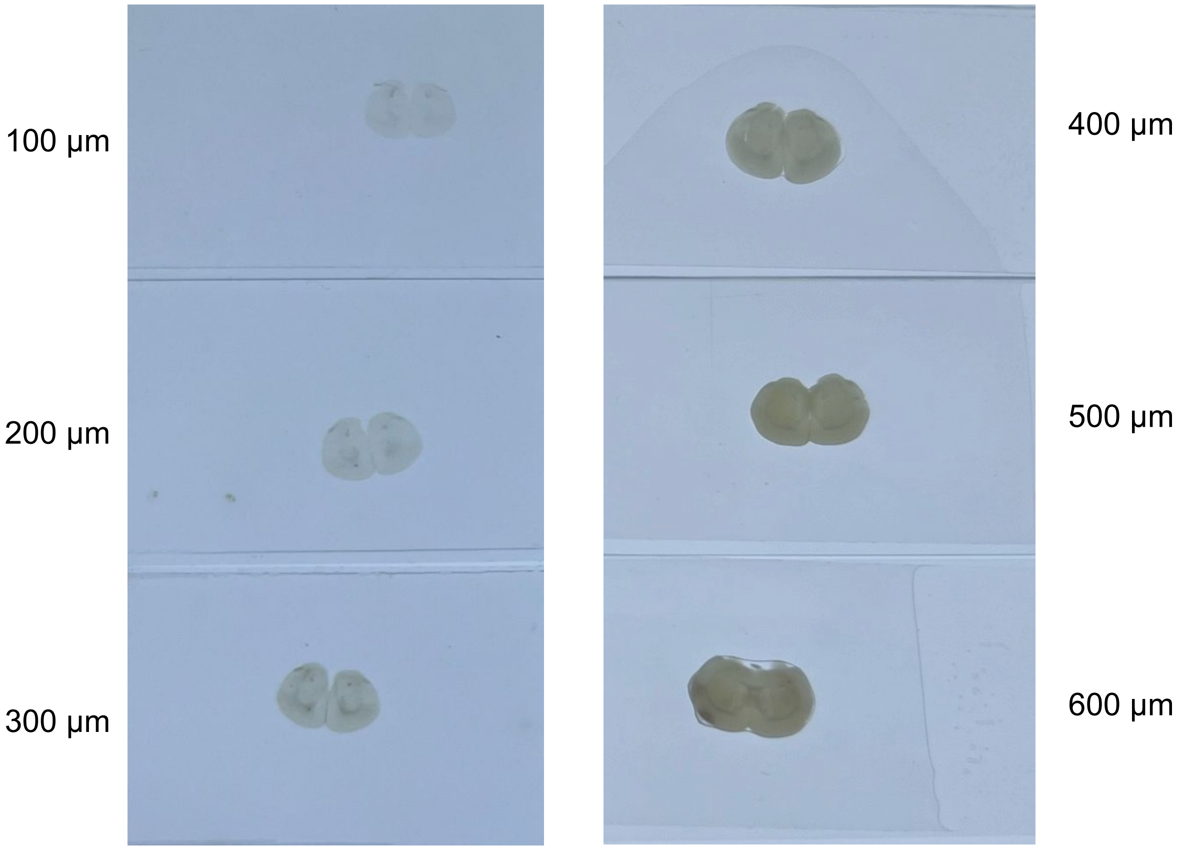

Fig. S2 The mouse brain slices with different thickness, ranging from 100 μm to 600 μm.

Supplementary Note 4 The principle of HDU-AO

Calibration between DM and SH-WS. The system aberration, which results from the misalignment of lenses, was compensated before the DM-SH-WS calibration. This aberration was pre-compensated using the methods outlined in Reference[4]. The measured results showed that the peak-to-valley (PV) value of the aberration was lower than 0.1 μm, which did not cause any signal and resolution degradation. Consequently, this aberration was disregarded in subsequent steps.

The calibration process between the DM and the SH-WS aimed to obtain a transmission matrix, denoted as M_{SH2DM}. This matrix establishes a relationship between spots shift in SH-WS and the actuator movement in DM. Initially, a flat mirror was positioned at the back pupil plane of the objective, thereby linking the DM and the SH-WS in the laser path. As a result, the DM, the pupil plane, and the lens array plane of SH-WS are mutually conjugated. Subsequently, the DM flat command was applied to generate a plane wavefront, which in turn generated a reference spot array in SH-WS. The spot locations of the array are represented as $S_{ref}= (x_1\ldots x_N, y_1\ldots y_N)^T$, where x_i and y_i denote the centroid location of the spot i. Third, 5000 DM test patterns, encompassing single-axis changes and multiple-axis changes with Zernike patterns, were recorded along with their corresponding spot locations in the SH-WS. The movements of DM actuators for each patterns were denoted as $\Phi=(a_1,a_2,\ldots,a_{140})^T$, where a_k is the normalized movement of actuator k. The spot locations of SH-WS for these patterns were denoted as $S = (x_1\ldots x_N, y_1\ldots y_N)^T$. Lastly, M_{SH2DM} was solved from Eq (1) using the least-squares method:

$$\boldsymbol{\Phi} = \boldsymbol{M}_{SH2DM}(\boldsymbol{S} - \boldsymbol{S}_{ref}), \quad (1)$$

Where Φ represents the movements of actuators of test patterns; S represents the spot locations of SH-WS corresponding to the test patterns.

Note that, M_{SH2DM} was used to calculate the aberration of the fluorescence. Hence, the system must be carefully calibrated to ensure the laser that reflected from the back pupil of the objective have the same emit angle and the same size as the fluorescence that collected from the focal point of the objective[5].

Sample aberration measurement. Prior to conducting the sample aberration measurement, it is imperative to acquire a fluorescent SH-WS reference, denoted as $S_{F\text{-}ref}$, from a sample that is devoid of aberrations. Under ideal circumstances, the laser reference spot diagram (S_{ref}) and the fluorescence reference spot diagram ($S_{F\text{-}ref}$) should be congruent. This congruence is crucial as we employ the transmission matrix (M_{SH2DM}), which is measured by the laser, to compute the wavefront aberration of fluorescence. Nevertheless, due to factors such as system chromatic aberration, there exists a pixel offset ranging from 0 to 5 between the two diagrams. Consequently, it becomes necessary to measure the fluorescence reference point diagram ($S_{F\text{-}ref}$) independently.

During the process of measuring the sample aberration, the laser was scanned within a confined FOV (25 μm × 25 μm). An SH-WS background was obtained by turning the objective lens switch off (no light passing through the objective), and subsequently, a fluorescent SH-WS image was captured by turning the objective lens switch on. The subtraction of the background from the SH-WS image aids in the suppression of ambient noise. Following this, the spot locations of the background-subtracted SH-WS image, denoted as S_F, were calculated by the centroid algorithm. Finally, the actuator movements of the DM, represented as ΦF, could be computed as Eq. (2):

$$\boldsymbol{\Phi}_F = \boldsymbol{M}_{SH2DM}(\boldsymbol{S}_F - \boldsymbol{S}_{Fs-ref}) \tag{2}$$

Reference

(1) Li, Z.; Zhang, Y. An efficient and user-friendly method for the synthesis of hexagonal-phase NaYF4:Yb, Er/Tm nanocrystals with controllable shape and upconversion fluorescence. *Nanotechnology* **2008**, *19* (34), 345606.

(2) Wang, F.; Deng, R.; Liu, X. Preparation of core-shell NaGdF4 nanoparticles doped with luminescent lanthanide ions to be used as upconversion-based probes. *Nat. Protoc.* **2014**, *9* (7), 1634-1644.

(3) Pologruto, T. A.; Sabatini, B. L.; Svoboda, K. ScanImage: flexible software for operating laser scanning microscopes. *Biomed. Eng. Online* **2003**, *2*, 13.

(4) Liu, R.; Li, Z.; Marvin, J. S.; Kleinfeld, D. Direct wavefront sensing enables functional imaging of infragranular axons and spines. *Nat. Methods* **2019**, *16* (7), 615-618.

(5) Yao, P.; Liu, R.; Broggini, T.; Thunemann, M.; Kleinfeld, D. Construction and use of an adaptive optics two-photon microscope with direct wavefront sensing. *Nat. Protoc.* **2023**, *18*, 3732–3766.
